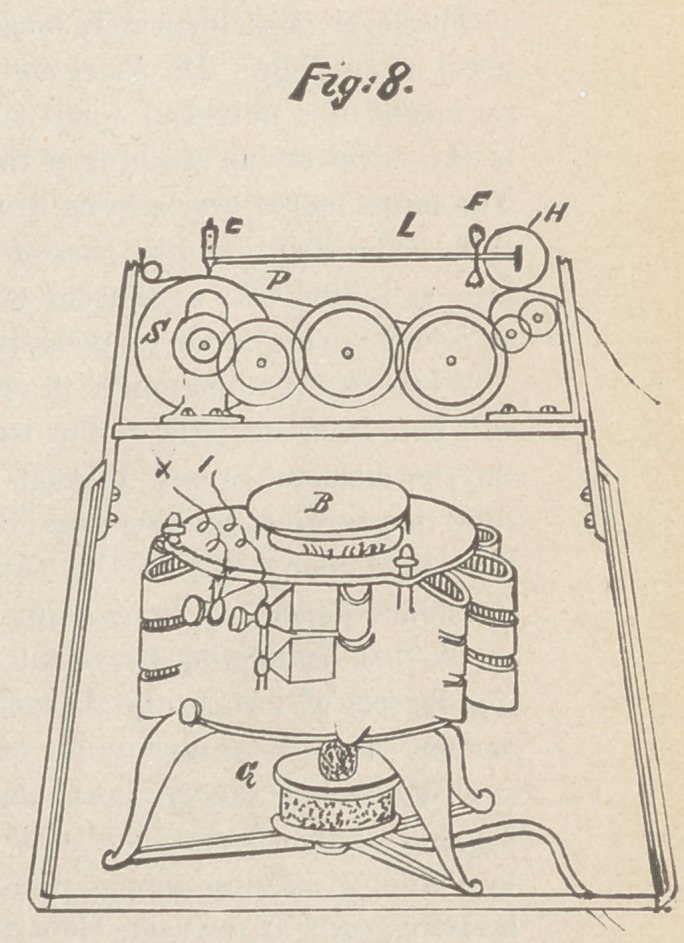# The Thermograph—Its Evolution and Destiny

**Published:** 1881-04

**Authors:** A. Wellington Adams


### THE THERMOGRAPH—ITS EVOLUTION AND DESTINY. By A. WELLINGTON ADAMS, M. D.

The introduction or application to medical science of no one instrument has afforded a more forcible impetus toward the goal of perfection, and the establishment of an exact science, than has the ushering in of the thermometer by Sanctorious, and subsequently the foundation, by de Haen, of medical thermometry.

And as the mind reviews and takes cognizance of the successively progressive steps constituting its history, from its inception to its present stage of development, one is compelled to look forward to disclosures far transcending present realizations.

Hence I was led, in the early part of 1879, to conclude that, notwithstanding the present degree of perfection in the science of medical thermometry, and the important function already performed by the thermometer in clinical, diagnostic, prognostic and experimental medicine, there was yet still further room for improvement. And, in fact, a palpable need for a more delicate and accurate thermometric system as an auxiliary to physiological, therapeutical and pathological investigations.

At present the office and usefulness of the system is sadly limited by its imperfections and narrow range of application. There are, no doubt, a few who will, upon first impulse, stamp this as a bold assertion, tending to depreciate the value of this most important aid to modern therapeutics, and, indeed, to all branches of scientific medicine. Such a *petitio principii* cannot, however, for one moment be allowed, since it must be acknowledged by all, that with existing appliances we are but able to ascertain the morning and evening minimum and maximum of temperature, regardless of the momentary or even hourly remissions and exacerbations of minor waves which fall within these, as it were, medium tidal points. By this latter expression I would have you understand me as referring to the points representing the morning and evening thermometric observations, A A A, fig. 1.

Now, a visual revelation of these constant thermal changes would, even with our present degree of knowledge concerning the pathognomonic character of certain thermal phenomena, be of incalculable value, and, therefore, a consummation to be devoutly sought for. But, outside of the advantages accruing from the application of existing knowledge to these observations, the anticipation of new developments and additional advantages resulting therefrom, becomes extremely plausible and justifiable; for it is as probable as it is possible that these momentary perturbations or minor waves, of which we as yet know almost nothing, are also conformable to law; forming, in different diseases, and under the administration of various drugs, characteristics quite as significant as do these medium waves, if I may be allowed the expression, when looked at collectively as preserved for a consecutive number of days.

From the results of these observations, taken, according to the present method, at comparatively irregular intervals, we can crudely construct a major wave, B B, fig. 1, analogous to the now so-called "fever curve," which may after a week or so prove characteristic of some peculiar form of the exanthemata, which we have become accustomed to recognize by comparison with a typical curve or major wave, whose conformation depends upon the position of these medium tidal points—that is to say, the points recording the morning and evening thermometric observations taken each successive day from the stage of pyrogenesis to that of defervescence.

I, therefore, concluded that in order to render the system more perfect and extend its range of application, it would be necessary to devise some means for procuring a thermometric record which should represent the *constant* condition of the subject under experimentation or treatment, for any desired length of time; this record to assume the form of a continuous curve automatically inscribed *pari passu* with the development of the disease.

This, of course, I found impracticable with the instruments already at the command of the profession. A solution of the problem, then, evidently depended upon the invention of an instrument capable of fulfilling these requirements. No sooner did this proposition present itself than I began to turn my attention in that direction. And is it to be wondered at, that in looking for the *principia* upon which would depend the construction of such an instrument, I should follow the footsteps of Breschet and Becquerel, who laid the corner-stone of the science by the aid of delicate thermo-electric apparatus? On the contrary, it is quite natural, that in looking for further boon, I should turn toward that force which has already been the means of marvelously facilitating commercial intercourse—of conferring upon man untold benefits of general application and lasting endurance—and of heaping upon science and its unfolders immeasurable *éclat.*

Having already given much attention to experimental electricity and acoustics, as bearing upon otology and laryngology, I was peculiarly and specially prepared, both as regards practical or detail knowledge, and the possession of suitable apparatus, to undertake the solution of so trying and complex a problem.

So, after adding to my laboratory much improvised paraphernalia for experimental research, I began operations, which, as before intimated, at first lay in the direction of thermo-electricity; to which principle I ascribed the ability to meet the requirements indicated in the first steps, *i. e.*, the contriving that portion of the embryo instrument capable of perceiving and responding physically to all variations in temperature with which it might be brought in contact, be they ever so slight. And, as regards this specific property, I was not in the least disappointed as to the inherent power of this principle; for, I procured several cylindrical tubes of antimony and bismuth, 3-16 of an inch in external diameter, and cutting them up into segments one-quarter of an inch long, then soldered these pieces together in alternate order, and lastly bent the whole into a spiral one-inch in diameter, to each end of which was attached a copper wire—one being fastened to a segment of bismuth at one end, and the other to one of antimony at the opposite end. I had then an instrument (see fig. 2) which would, when placed in the axilla, and the terminal wires connected with the binding posts of a differential galvanometer, give evidence, through the varying deflections of the galvanometer needle, of the most minute and transient thermal fluctuations.

This, however, was not the only indication to be met. There was the recording part yet to be constructed, and that necessarily must bear a certain relationship to the thermometer proper.

In the device described above and figured in illustration No. 2, there were generated thermo-electric impulses sufficiently strong to induce an extensive range of deflections in a sensitive galvanometer, but entirely too weak to manifest themselves in any more crude manner; while, of course, the delicacy of the galvanometer rendered impossible the application of mechanical appliances for graphically recording deviations; for it must be borne in mind that with the graphic method, the first essential is a surface traveling in a straight line at a uniform rate of speed; this may be accomplished after any of the known methods. The second essential consists in the provision of an automatic writer for inscribing the record upon said surface. This, as is generally the case, may be effected by means of a delicate lever, having attached to its longer extremity a marker so arranged as to move backward and forward over the traveling surface. When the lever is quiescent and the movable surface in motion, a horizontal line is made, known as the *abscissa*. This signifies time, and all deviations from this line are called *ordinates*. These may vary in direction, from the vertical, to any degree of obliquity, according as the impingements upon the shorter end of the lever, which give rise to them, are abrupt or sluggish.

It will readily be seen, then, that with the addition of these pieces of mechanism, we have a commensurate amount of friction to contend with, and in order to overcome this, our electric impulses must be stronger than could be generated by the small antimony and bismuth spiral seen in fig. 2.

Not in the least daunted, however, by the dissipation of all hopes in this direction, I still continued my researches, fully believing that even many more failures were in store for me, as the *res necessariæ* to the proper evolution of a successful issue.

Experience seemed now to clearly indicate a necessity for a definite source from which to derive a constant and unvarying supply of electricity, of sufficient strength to undergo a mechanical metamorphosis equal to our requirements. This current, however generated, should be moulded into electrical waves corresponding to the thermotic changes, through the introduction, at any given point in the circuit, of some physical or chemico-physical device capable of accomplishing the same. To effect this, the instrument illustrated in fig. 3 was constructed. This consists virtually of an ordinary thermometer made of vulcanite and bent into a spiral. For convenience of illustration this shape is not shown in the cut. The bulb was lined with a silver film in electrical communication with the lower binding-post, A. Up both sides of the shaft B, and at right angles to its axis, minute channels were drilled at uniform distances from each other, and leading into the shaft bore. These channels, instead of being placed opposite each other, were arranged one above the other, *e. g.*, the first one on the right hand side (C) being placed 1-32 of an inch from the first one on the left hand side (D), while the *second* one on the right hand side (E) was placed 1-16 of an inch from C, and 1-32 of an inch from D; and so on throughout the entire series. The shaft-bore was now temporarily filled with a wire of corresponding caliber, and the aforesaid channels filled with melted platinum. A No. 36 silk-covered copper wire was next soldered to the first platinum *fibrillus* (C), and then coiled several times around the shaft (B), finally to be soldered at its terminal extremity to the platinum *fibrillus* (D). Another corresponding piece of wire of like length, caliber, etc., was soldered to the platinum *fibrillus* (D), and now an equal number of lamillar convolutions made around the shaft (B), and its terminus soldered to the platinum *fibrillus* (E). When all these *fibrilli* were treated in like manner, and the last wire in the series connected with binding-post (F), a vacuum formed, and the mercury inserted in the bulb, the instrument was complete, and only awaited a trial to prove its efficacy or inefficiency, as the case might be. To an electrician the *modus operandi* of this apparatus becomes at once apparent. The binding-posts A and F being placed in an electric circuit and the thermometer bulb subjected to thermal variations—the instrument is in operation. Each of the above described coils of wire goes to form a series of "resistance coils," and any number of these are introduced into the circuit or "short-circuited"—that is, cut out, and the strength of the current thus diminished or increased, by the rising and falling of the column of mercury. For, when the column of mercury rises to the first platinum *fibrillus* (C), the electric current will pass from the binding-post (A) through the column of mercury to the first platinum *fibrillus* (C), and thence through all the "resistance coils " to emerge at the binding-post (F), on its way to complete the circuit. If, however, the column of mercury rises to the *fibrillus* (D), the first "resistance coil" is short-circuited, and the current is obliged to pass through the remaining coils *only*. Thus the resistance is diminished a certain number of ohms and the current accordingly strengthened a relative amount of volts. This instrument was experimentally a complete success, inasmuch as with it there could be produced electrical impulses varying in intensity in accordance with the character of the temperature changes brought in contact with it. Moreover, the current designed to be thus moulded into waves could have any desired electro-motive force, and this, as before explained, was a *sine quâ non*.

When it came to the question of a practical application, though, this instrument, also seemed to be deficient in many respects. The principal defect lay in the fact that the electric waves were not *always* in exact relationship with the thermometric indications. For, supposing the column of mercury to have reached a point half-way between C and D, the electricity would then meet with a resistance equal to that offered when the column stood no higher than C, hence we should have no increased strength in the current to mark this difference in temperature. Another thing rendering it somewhat impracticable, was its complicated nature and expensiveness.

My researches now remained in *statu quo* for some time, when one day, in the course of conversation with Dr. J. Harry Thompson, of Washington, D. C., he suggested the utilization of the newly discovered property of carbon to vary its conductivity under different degrees of pressure.

I immediately availed myself of this suggestion, developing the instrument as now perfected and illustrated in figs. 4 and 5. This is the thermometer proper or responding portion of the instrument, and consists of a spiral spring made of two *lamellæ* of brass and steel respectively, soldered together, the brass occupying the outer side. Of course this spring expands uniformly with equal increments of heat, and the brass, the most expansible of the two metals, will, upon a rise of temperature, give the platinum knob (a), attached to the free end of the spring, a concentric twist. In this way we produce a varying pressure upon the contents of the vulcanite tube (T), against which the platinum knob (a) impinges.

The other end of the substance contained in the hard-rubber tube (T) has for its abutment the platinum knob (b) attached to the hard-rubber bracket (C). The whole. as seen in fig. 4, is inclosed in a perforated German-silver case, with rounded edges, and having an external diameter of but 1¼ inches.

The binding-post (A), fig. 4, is in electrical communication with the platinum knob (a), and the binding-post (B) is in electrical communication with the platinum knob (b). When the apparatus is introduced into an electric circuit, by attaching the two poles to the two binding-posts, the current enters through one and emerges at the other, passing in its course, through the substance in the vulcanite tube (T). The two little handles (H) (H) are intended as a means of securing the instrument in its proper position in the axilla. The composition used in the vulcanite tube (T) may be either a solid stick of baked lamp-black, a series of carbon discs with intervening ones of silver, or a powder made of plumbago, gas-carbon and silver, finely divided. After receiving a communication from Thos. A. Edison in regard to this matter, I commenced a series of experiments to determine the most suitable composition for this purpose, and the best results were obtained from the powder already referred to. The salient feature of this instrument is the changing of its electrical resistance with pressure, and the ratio of these changes, moreover, corresponding exactly with the pressure, the latter, in turn, being dependent upon and in unison with the rise and fall of temperature.

Here, then, was the true solution, for, by subjecting this instrument to varying degrees of temperature, the resistance of the powder would vary in precise accordance with the pressure exerted by the uniform expansion of the spiral spring under equal increments of heat, and consequently a proportionate variation would be produced in the strength of the current. The latter would thus possess all the characteristics of the heat waves, and by its reaction through the medium of some electro-magnetic piece of mechanism yet to be devised, these might be transferred to our

A.A.A.- Medium tidal points. B.B.- Major Wave. D.D.D.- Medium Waves.

C.C.C.- Intervals in which the minor waves occur.

Fig. 2.

Fig. 3.

G- Galvanometer. S- Battery. H.H.H.- Coils in position.

R.R.- Diagram showing arrangement of coils.

S- Silver film lining bulb.

Fig. 4.

Fig. 5.

Fig. 5- Inside plan Fig. 4.

H.H.- Handles.

A and B- Binding posts.

T- Vulcanite tube.

a and b- Platinum knobs.

movable surface, in the form of a sinuous line, whose rising and falling inflections would give a graphic representation of them.

Now, that I had satisfactorily reduced this portion of the problem, the next in order was the devising that part of the instrument intended for recording such variations as the other branch might be subjected to. This, I assure you, was no easy task, but one requiring a mint of patience and tedious application. For, first—it must be simple; second—there must be established a permanent relationship between the first and second branches of the instrument; in other words, there must exist throughout a strict interdependence; third—in order that the electro-motive force required might be reduced as much as possible, it must be delicate; fourth—to render the latter possible, friction must be practically reduced to a minimum. To carry you through the almost endless and varied experiments necessary in developing means for meeting these indications would be as tiresome as it would be unnecessary. Hence, I shall confine myself to the result only.

If a number of coils of insulated wire be wound around a hollow reel, there is formed what is known to electricians as a *helix*. If this is now placed in an electric circuit and a current passed through its convolutions, it is temporarily constituted a magnet, the two ends forming the poles; so that it may be said to possess all the properties of a permanent magnet during the passage of the current. Moreover, if such a helix, mounted in a vertical position in such a way that an iron rod can be introduced into it from below, be connected with a battery, the iron rod will be at once drawn up into it and be sustained oscillating in its axis, even though the rod may weigh considerable.

The depth the iron rod enters will also depend entirely upon the strength of the current and the amount of resistance offered by the iron rod. This principle is well-known in physics as the "axial electro-magnetic force," and in it I found what I sought, namely—a combination of delicacy and strength in the proper proportions. A diagramatical illustration of its application may be seen in fig. 6, where H represents a helix of peculiar construction applied to the purpose in hand; D is a soft iron tube in connection with the short end of the lever (L); P, represents the

Fig: 6.

Fig: 7.

Fig: 8.

movable surface or strip of paper; F, the fulcrum of the lever, and E its marker or stylus; T, the thermometer proper introduced into the circuit; X, the battery; C, the curve, and S, the brass drum over which the strip of paper (P) moves.

Having comprehended the principles, the action of this combination is obvious. If an electric current passes through the helix (H), the core (D) will be drawn into said helix, carrying with it the short end of the lever (L), to which it is attached. This movement naturally causes the marking end of the lever to make a still longer excursion in the opposite direction. Upon breaking the circuit, the attractive power of the helix is abolished, and the counter-action of the spring (s) returns the lever to its normal position.

The depth to which the core (D) is drawn into the helix (H) being dependent upon the strength of the current passing through the coils of wire, the excursions of the tracer or lever will also be great or small, according as the current is weak or strong.

The lever (L) is delicately made, and its fulcrum provided with jewel mountings. Its short end is connected with the core (D) by means of a universal joint, while its longer end has inserted in it a silver stylus reaching to the surface of the traveling paper. The latter moves over a brass drum forming a portion of the circuit. The strip of paper passing over the brass cylinder, having been saturated with a solution of chloride of sodium, pyrogallic acid, and ferrocyanide of potassium, the instrument is complete.

When this combination is in operation, a current of electricity will pass from one pole of the battery to the binding-post (A) of the thermometer proper, through the substance in the vulcanite tube to emerge at binding-post (B); thence through the helix to the lever, along this to the silver stylus; thence through the moistened paper and brass cylinder to the other pole of the battery—thus completing the circuit. Upon the application of varying degrees of heat to the thermometer proper, the resistance the current meets with during its course will be varied in precise accordance with the various changes of temperature. This waxing and waning current will now pass through the helix, and by the latter's peculiar action produce to and fro motions in the lever, passing, at the same time, through the lever and chemically prepared paper, and producing as it passes a double chemical decomposition upon the paper; one of which decompositions renders the development of friction, during the movements of the lever, so slight as to be imperceptible; the second decomposition producing a change in color upon the paper, corresponding to the movements of the stylus, and affecting no larger surface than it covers, thus obviating the additional friction accompanying the use of an ordinary maıker.

From this description you will understand that the lever is moved backward and forward by a difference in the attractive power exerted by the helix, this in turn being dependent upon the strength of the current, which has already passed through the thermometer proper, and there been moulded into electric waves corresponding to the heat waves; the motion of the lever being facilitated by the lubricating action of the current, as the result of one of the chemical decompositions during its passage through the chemically moistened paper; while the other decomposition causes a discoloration, and thus produces a mark corresponding in outline to the movements of the lever. This mark will, therefore, form an irregular line, whose sinuosities will give a graphic representation of the heat variations. This apparatus is extremely sensitive and can be made to record 1-100 of a degree.

Now, after marking upon our strip of paper the minimum and maximum points representing respectively 90° and 110°, it becomes a very easy matter to determine the degree of heat represented by any point lying within this range. This is accomplished by dividing the intervening space into any number of equal parts, when any one of these divisions will represent a degree or any part of a degree, according to the number of divisions. These horizontal lines may be placed at such distances from each other as to represent 1-10 of a degree. Having provided the traveling paper with a uniform speed, it also becomes an easy matter to determine the time represented by any given distance upon its surface; for, supposing a certain amount of paper passes a given point in the instrument in one hour, to determine the amount passing the same point in five minutes, it is only necessary to draw vertical lines dividing this distance into twelve equal parts, each one of these will then represent five minutes.

After determining upon the principles it becomes very easy to work up the details that would place the instrument in a convenient form for manufacture and use. These may be seen, as applied, in figs. 7 and 8. Fig. 7 is a front elevation of the complete thermograph.

It consists of a cast-iron case having two departments, one for the recording mechanism and actuating clock movement, the other for the battery. In the upper part of the front there is a circular depression for the reception of the thermometer proper or perceiving portion of the instrument when not in use.

This is held in place by means of two little catches, one on either side, as seen in the figure. On both sides of this are the binding posts for the reception of the wires leading from the thermometer proper when the latter is in position in the axilla. The open work in the lower portion is intended for the ingress of air and egress of gases. Fig. 8 is an interior elevation with the front removed; above is seen the recording mechanism, and below the thermo-electric battery. This form of battery gives a continuous and unvarying current, requires no cleaning or recharging, and costs but little to run, hence it is the most available source from which to derive the current; the heat for operating it may be supplied by either an alcohol lamp or a gas-burner. Not only is it possible with this instrument to procure a continuous curve denoting the constant febrile condition of a subject, but, with the addition of certain accessories now in process of construction, and as suggested by Prof. Mayer, of the University of Technology, Hoboken, and Dr. Toner, of Washington, we may be able to procure, on the same strip of paper, at the same time and under similar conditions, a sphygmographic and a respiratory curve; thus enabling pathologists, therapeutists, physiologists, and, in fact, general practitioners, to study the inter-relationship of these three cardinal symptoms under various modifying circumstances. These are the *possibilities*, but when we drift into the *probabilities*, we see in prospective the addition of that which will also furnish a moisture curve. Of the advantages of the graphic method as applied to medicine, I need hardly speak. It already promises for medicine what it has accomplished in physics.

Every physicist adores such familiar names as Leon Scott and Dr. Clarence Blake, to whom we are indebted for the application of the graphic method to the science of acoustics, through the medium of the phonautographs invented by themselves.

To the experimental therapeutist this instrument is of incalculable value, as affording a means of determining the precise character of the temperature changes under the administration of various therapeutic agents in different sized doses and modes of exhibition.

The experimental physiologist will find in it that which will materially facilitate accurate observation in his field. And the advantages accruing from its application in pathological investigations, and the possibility of thus elucidating hitherto obscure phenomena, must be patent to every one. An instrument of so much value as an aid to observations in these three important branches of scientific medicine, needs no further lauding; but I cannot draw my paper to a close without setting forth the mode of application and the advantages attending its use in every day practice.

Take, for example, a suspected case of typhoid fever, experience and experimentation with the thermograph having already revealed a characteristic *minor* wave curve for typhoid fever. The physician is summoned. Upon arriving he applies his thermograph in the following manner: First, the perceiving portion, as seen in fig. 4, is fastened in the axilla by means of two elastic bands attached to the handles H H, one passing around the trunk, the other over the shoulder. Next, two fine and flexible silk covered wires are led from the binding-posts A B, fig. 4, to the binding-posts B B, of fig. 7, the latter having been previously placed upon a stand at the head of the patient's bed.

The wires, of course, are of sufficient length to admit of any degree of motion on the part of the patient without interfering with the position of the recorder. The instrument will now be ready for use, and, upon starting the battery, it will continue in operation for any desired number of days, with little or no attention outside of winding and replenishing with new rolls of paper.

The first benefit to be derived from its use in such a case, consists in the ability to determine upon a diagnosis much earlier than would ordinarily be possible; second, the physician is furnished with a permanent record of the condition of his patient from hour to hour and day to-day; third, the slightest modification or variation by reason of an exposure, the exhibition of prescribed remedies at given hours, or the ingestion of prescribed food during the day, will be revealed to the physician when he makes his evening visit, thus affording him from time to time, a more definite idea of the immediate effect, good or bad, of his treatment: fourth, it will give warning of danger from collapse during the crisis before it could be detected in any other way; fifth, the physician is provided with a means of leaving more definite directions with the attendant or nurse, *e. g.*, he will be able to say that "should the curve assume such or such a character, or the line rise to this or that point, you may discontinue this, that, or the other remedy, and proceed to exhibit *this*, according to the directions; or, should such and such a thing take place it will indicate an emergency calling for this, that, or the other measure."

The science of meteorology, also, will find in the thermograph an instrument it has long felt the need of. Never before has there been invented an instrument capable of furnishing a curve representing the constant temperature of the atmosphere. To be sure, there are two or three instruments in the possession of the United States Weather Department at Washington, constructed upon an entirely different principle, which automatically produce a continuous curve, but the latter is only by reason of the velocity with which the cylinder revolves, besides, they are of an exceedingly complicated nature, cumbersome, and *very* expensive.

The simplicity and inexpensiveness (it will cost about $50) of the thermograph, places it within the reach of almost every physician, and will enable the United States Weather Department to furnish one or more of them to every one of its sub-stations.

This, gentlemen, is the instrument I have chosen to dignify with the title of *Thermograph*, and which I have lately placed in the hands of Aloe & Hernstein to manufacture for the use of the medical profession.

With its introduction, I predict the dawn of a new era in medicine, marked by progress equal to that accompanying the introduction of the sphygmograph, myograph, cardiograph, and other important instruments of a similar character.

[Condensed from a paper read before the El Paso County Medical Society, Colorado Springs, Colorado.—*Rocky Mountain Medical Review.*]

---

A COMMITTEE has been appointed by the New York Academy of Medicine, consisting of Drs. H. G. Piffard, F. R. Sturgis and G. H. Fox, to investigate the extent to which leprosy prevails in the United States. We are glad to note that this subject is attracting the attention which it deserves. A committee of the American Dermatological Association, a national body, has had this subject in charge for several years and has regularly published the statistics which it has laboriously accumulated. Its chairman is Prof. James C. White, of Boston, the eminent dermatologist of Harvard University, and probably the oldest dermatologist in this country. Physicians knowing of the existence of lepers in any part of the United States, are requested to communicate with these gentlemen. The address of Prof. White is No. 10 Park Square, Boston, Mass.

COOK COUNTY HOSPITAL.—On April 1, Dr. G. F. Bradley was put in charge of the Medical wards, replacing Dr. B. C. Gudden. Dr. L. L. McArthur became House Surgeon, taking the place formerly occupied by Dr. W. P. Verity. Dr. B. C. Meacher is first assistant to Dr. McArthur, Dr. H. Kendall occupies the same position on the Medical side.

## Figures and Tables

**Fig.1. Fig.2. Fig.3. Fig.4. Fig.5. f1:**
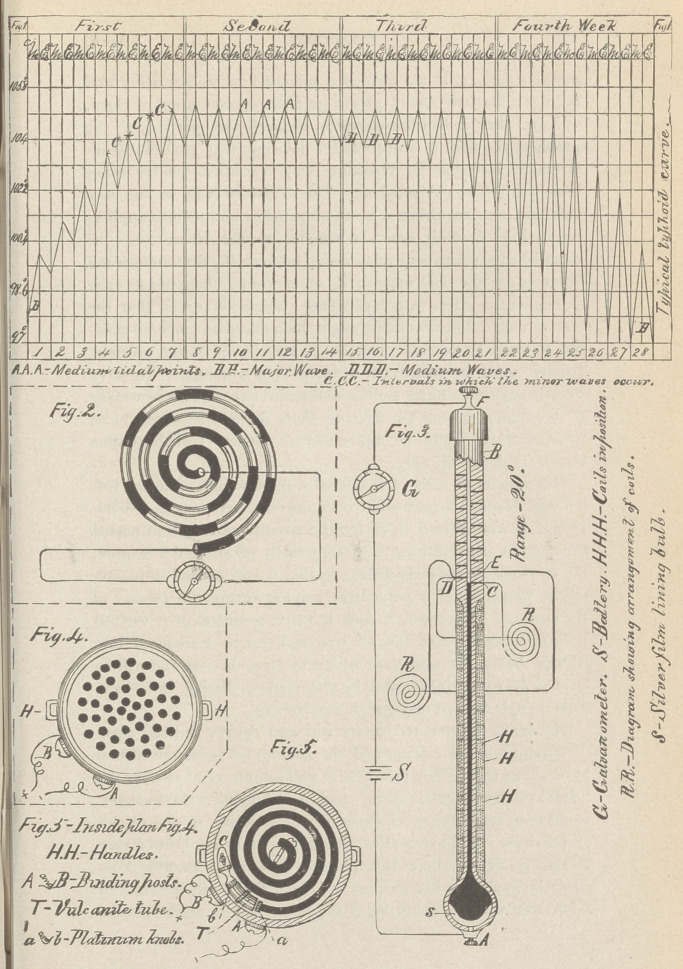


**Fig:6. f2:**
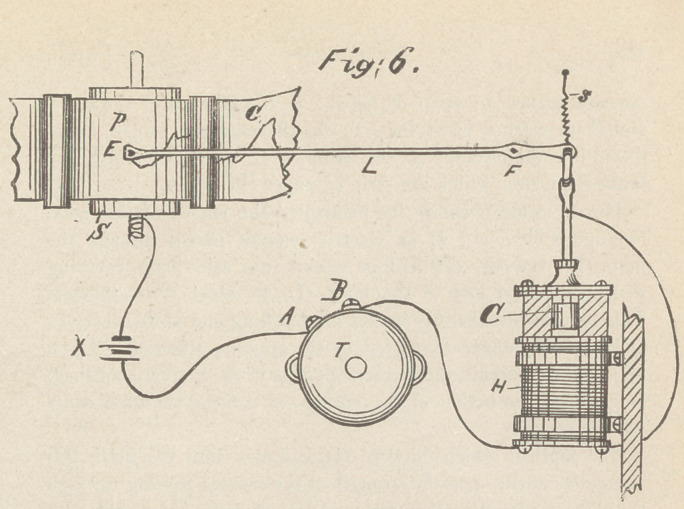


**Fig:7. f3:**
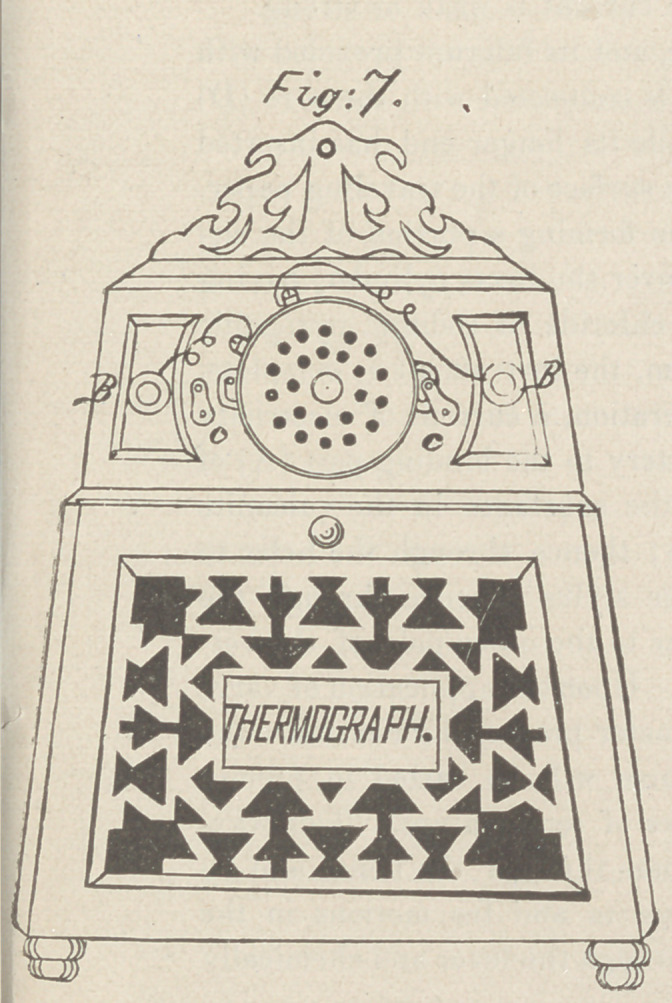


**Fig:8. f4:**